# Remote Monitoring of Chemotherapy-Induced Peripheral Neuropathy by the NeuroDetect iOS App: Observational Cohort Study of Patients With Cancer

**DOI:** 10.2196/65615

**Published:** 2025-02-05

**Authors:** Ciao-Sin Chen, Michael P Dorsch, Sarah Alsomairy, Jennifer J Griggs, Reshma Jagsi, Michael Sabel, Amro Stino, Brian Callaghan, Daniel L Hertz

**Affiliations:** 1 Department of Clinical Pharmacy University of Michigan College of Pharmacy University of Michigan Ann Arbor, MI United States; 2 Department of Internal Medicine University of Michigan Medical School Ann Arbor, MI United States; 3 Michigan Medicine University of Michigan Rogel Cancer Center Ann Arbor, MI United States; 4 Department of Radiation Oncology Emory University School of Medicine Atlanta, GA United States; 5 Department of Neurology University of Michigan Medical School Ann Arbor, MI United States

**Keywords:** chemotherapy-induced peripheral neuropathy, smartphone, mobile health, gait, balance, manual dexterity, mobile phone, chemotherapy, peripheral neuropathy, CIPN, neurotoxic chemotherapy, monitoring, detection, patient-reported outcomes, mHealth, application

## Abstract

**Background:**

Chemotherapy-induced peripheral neuropathy (CIPN) is a common and debilitating adverse effect of neurotoxic chemotherapy characterized by symptoms such as numbness, tingling, and weakness. Effective monitoring and detection of CIPN are crucial for avoiding progression to irreversible symptoms. Due to the inconvenience of clinic-based objective assessment, CIPN detection relies primarily on patients’ reporting of subjective symptoms, and patient-reported outcomes are used to facilitate CIPN detection. Our previous study found evidence that objective functional assessments completed within a smartphone app may differentiate patients with and those without CIPN after treatment.

**Objective:**

This prospective, longitudinal observational cohort study aimed to determine the feasibility and accuracy of app-based remote monitoring of CIPN in patients with cancer undergoing neurotoxic chemotherapeutic treatment and to conduct exploratory comparisons of app-based functional CIPN monitoring versus patient-reported outcome–only monitoring.

**Methods:**

The NeuroDetect app (Medable Inc) includes subjective EORTC (European Organization for Research and Treatment of Cancer) Quality of Life Questionnaire (QLQ)–20-item scale (CIPN20) and 6 objective functional assessments that use smartphone sensors to mimic neurological examinations, such as walking, standing, and manual dexterity tests. The functional assessment data were collected from patients with cancer undergoing neurotoxic chemotherapy, and a neurological examination was conducted at the end of treatment to diagnose CIPN in the feet (CIPN-f) or CIPN in the hands (CIPN-h). Various classification models including NeuroDetect features only (NeuroDetect Model) CIPN20-only (CIPN20 Model) or a combination of both (Combined Model) were trained and evaluated for accuracy in predicting CIPN probability.

**Results:**

Of the 45 patients who completed functional assessments and neurological examinations, 24 had CIPN-f, and 29 had CIPN-h. The NeuroDetect Model could discriminate between patients with and those without CIPN-f (area under the curve=83.8%, comparison with no information rate *P*=.02) but not CIPN-h (area under the curve=67.9%, *P*=.18). The rolling rotation features from the eyes-closed phase of the Romberg Stance assessment showed the greatest contribution to CIPN-f (40% of total variable importance) and the Finger Tapping assessment showed the greatest contribution to CIPN-h (85% of total variable importance). The NeuroDetect Model had numerically, and at some time points statistically, superior performance to the CIPN20 Model in both CIPN-f and CIPN-h, particularly before and early in treatment. The Combined Model numerically, though not statistically, outperformed either assessment strategy individually, indicating that the combination of functional and patient-reported assessment within a smartphone may be optimal to CIPN detection.

**Conclusions:**

Our findings demonstrate the feasibility of integrating subjective and objective CIPN assessment into a smartphone app for remote, longitudinal CIPN monitoring. Studies of larger patient cohorts are needed to refine the app-based CIPN detection models and determine whether their use in practice improves CIPN detection.

## Introduction

Chemotherapy-induced peripheral neuropathy (CIPN) is a common adverse effect of chemotherapy, affecting up to 70% of patients receiving taxanes for breast, lung, ovarian, and other cancer types [1]. CIPN typically presents with numbness or tingling in the feet or hands that may progress to motor weakness and impairment [2,3]. In some cases, CIPN can be irreversible, resulting in long-term gait imbalance and impaired manual dexterity, which hinder daily activity, reduce independence, increase fall risk, and reduce quality of life [4,5].

Detecting CIPN during treatment is critical to avoid these potentially irreversible adverse effects [4,5]. Detection of CIPN during neurotoxic chemotherapy treatment primarily depends on patients self-reporting symptoms during routine appointments with their medical oncology team [6]. Objective CIPN assessment may be useful to detect symptoms but is not commonly used in practice due to logistical barriers, including the need for additional trained personnel, time, space, and equipment [7]. As an alternative, validated patient-reported outcome (PRO) questionnaires have been developed to enable convenient subjective CIPN assessment. One such tool is the CIPN Quality of Life Questionnaire developed by the European Organization for Research and Treatment of Cancer (EORTC), which includes a 20-item scale (CIPN20) [8]. PROs may be an improvement over routine physician assessments via the National Cancer Institute Common Terminology Criteria for Adverse Event grading, but PROs lack objective measurement and rely on patients’ willingness to accurately report CIPN symptoms [9]. Consequently, there remains an unmet need to develop strategies for objective CIPN assessment that are feasible and accurate.

Smartphone and wearable sensor-based technology have been increasingly used to advance the detection and monitoring of various diseases [10], for example, smartphone apps can monitor gait and balance changes and provide valuable insights for patients with progressive Parkinson disease [11]. Similar to Parkinson disease, objective evidence of CIPN includes impaired gait, increased sway, and reduced manual dexterity, all of which can be detected through functional assessments using wearable or smartphone sensors [12,13]. Our previous study of the first version of NeuroDetect (Medable Inc) used off-the-shelf smartphone sensor–based functional assessments and showed feasibility in differentiating patients with and those without CIPN posttreatment [13]. Integrating PRO data with objective assessments within a smartphone app could achieve remote and objective CIPN monitoring and enhance the detection of CIPN with minimal inconvenience and cost [14]. The objective of this longitudinal study was to determine the feasibility of conducting longitudinal, remote smartphone-based functional assessments that emulate certain aspects of a formal bedside neurological examination and investigate whether these functional assessments can detect CIPN during neurotoxic treatment. Exploratory analyses in this unpowered feasibility study investigated the sensitivity and specificity of an app-based approach compared with or combined with PRO-only CIPN assessment.

## Methods

### NeuroDetect App

NeuroDetect is an iOS (Apple Inc) app that incorporates both subjective and objective evaluations of CIPN. The subjective evaluations include EORTC Quality of Life Questionnaire (QLQ)–CIPN20, a validated PRO questionnaire for the collection of CIPN sensory, motor, and autonomic symptoms on a 4-point Likert scale (1=not at all, 2=a little, 3=quite a bit, and 4=very much) [8,15]. The objective functional assessments were customized from the available activities in the ResearchKit (Apple Inc) framework to replicate certain aspects of the neurological examination [16]. The 6 NeuroDetect functional assessments use embedded sensors within the smartphone including an accelerometer, gyroscope, and touchscreen to monitor speed, sway, accuracy, and rhythm during gait and balance tasks (natural walk, tandem walk, tandem stance, and Romberg stance) and manual dexterity tasks (finger tapping and hole-peg test). For both natural walk and tandem walk, the user is asked to place their phone in their back pocket while taking 10 steps forward, turning around, and then walking 10 steps back at their usual pace [16,17]. For both tandem stance and Romberg stance, the user is asked to stand as still as possible for 10 seconds. In tandem walk and tandem stance, the user’s feet are lined up with 1 foot directly in front of the other while walking or standing, respectively [17-19]. In Romberg stance, the user stands still for 10 seconds with their eyes open and then for another 10 seconds with their eyes closed [18,19]. In finger tapping, the user taps 2 buttons on the screen of their phone with their index and middle fingers, alternatively for 100 taps or 100 seconds (whichever comes first) [16]. In the hole-peg test, which is adapted from the standard 9-hole peg test, the user repeats the process of placing a “peg” (indicated by a closed circle) into a “hole” (indicated by an open circle) on the phone screen and then removing it 4 times [13,20].

### Study Participants

Patients scheduled to begin taxane or platinum treatment for breast or colorectal cancer were identified by the Breast or Colorectal Clinical Research Teams within the University of Michigan Rogel Cancer Center. After the medical oncologist approved recruitment of the patient, the study team called the potential participant up to 3 times to describe the study and assess their eligibility and interest in participating. Patients who were interested in participating in the study were screened during the phone call to confirm that they (1) had consistent access to an iPhone (Apple Inc) and (2) did not have baseline neuropathy, defined as any score >1 in the first 4 items of the CIPN20, which ask about tingling or numbness in the fingers or toes [21]. After screening, interested participants completed verbal informed consent to participate and were provided email instructions to download NeuroDetect and complete the integrated written informed consent, a baseline CIPN20, and the 6 functional assessments.

Patients were asked to repeat the CIPN20 and functional assessments within 24 hours before each chemotherapy cycle. Depending on the chemotherapy regimen, the evaluations ranged from every 2 to 4 weeks and the timing was adjusted to accommodate treatment delays or early discontinuations. Patients were also allowed to complete additional evaluations during treatment.

Within 1 week before or 2 weeks after the end of chemotherapy treatment, patients underwent a structured clinical neurological history and examination that included assessment of neuropathic symptoms, signs, and reflexes, which was conducted by neuromuscular specialists (BC and AS) on the study team. CIPN signs and symptoms were diagnosed based on the Toronto Consensus Definition of possible clinically evident distal symmetrical polyneuropathy, which was considered the gold-standard CIPN definition for this study [22,23]. Each patient was clinically assessed for sensory symptoms (numbness, dysesthesias, hypersensitivity to touch, or pain), muscle strength, sensory function of small and large fibers, gait and coordination, and reflexes. From these data, the clinician determined whether the patient had symptoms, sensory exam findings, or decreased reflexes consistent with polyneuropathy. If at least 1 of these 3 were present, a patient is considered to have possible CIPN and thus was considered a CIPN case for this analysis. CIPN cases were then classified as experiencing CIPN in the hands (CIPN-h) or CIPN in the feet (CIPN-f) based on the location of the symptom or sensory or reflex exam finding that caused them to be considered a CIPN case.

### Data Preprocessing

Sensor data from the walking and standing samples were filtered and preprocessed before feature extraction. Walking forth and back were treated as 2 samples. Walking samples with durations of less than 7.5 seconds in natural walking or 10.5 seconds in tandem walking were discarded as these indicate the patient stopped the walking task rather than completing it. Walking samples were trimmed to keep only the active walking phase without interruption, including turning around. Walking and standing samples were rotated to the global reference frame to adjust for individual variations in the orientation of the patient’s phone in their pocket or the direction the patient faced while walking.

A total of 3700 features were generated from the 6 functional assessments (Table S1 in Multimedia Appendix 1). An open-source tool, mhealthtools in R (Posit), was used to extract statistical or informational features from sensor data of walking, standing, and finger-tapping assessments [24]. For walking and standing data in each of the 3 dimensions of acceleration (side-to-side, forward-backward, and up-and-down) and rotation (pitch, yaw, and roll), empirical 2-mode decomposition transformation and empirical wavelet transformation were used, respectively, to generate features in time-domain (signal amplitude and distribution) and frequency-domain (signal composition in different frequencies) [24]. For finger-tapping data, features of tapping intervals and drifts were generated to characterize both dominant and nondominant hand movements [24]. The mean and SD of the duration of placing and removing pegs were calculated for hole-peg tests in R.

Features were filtered using an ensemble method of minimum redundancy maximum relevance through mRMRe in R to account for multicollinearity [25]. The number of features to select was based on the number of features with a positive score, which means the relevance to the outcome was higher than redundancy with any previously selected features. Feature selection was determined by majority voting among multiple solution sets through 3 steps. First, baseline features were filtered within each assessment and voted among 5 solution sets. Second, end-of-treatment features were filtered together with selected baseline features within each assessment and voted among 5 solution sets to remove features correlated with the baseline. Third, all selected end-of-treatment features were further filtered and voted on among 15 solution sets to remove features correlated with each other between different assessments.

### NeuroDetect Model Training and Testing

In total, 2 elastic net classification NeuroDetect Models were built to filter feet and hand features, respectively, and to achieve the highest area under the curve (AUC) in detecting CIPN-h and CIPN-f [26]. NeuroDetect Models were trained using 70% of the end-of-treatment data set with repeated cross-validation through caret and glmnet in R, and the performance was evaluated in the unbiased 30% testing data set [27,28]. The numbers of repeats and folds were the square root of the sample size. Optimal model parameters were first searched using sequences automatically generated by the glmnet package based on the range of input data, which were then fined-tuned to the same magnitude [28]. Infinite values were imputed with 1.5 times the maximum value. All input values were centered and scaled before training and testing. Variable importance was calculated using the caret package based on regression coefficients [27].

NeuroDetect Model performance was evaluated via AUC and accuracy, which was compared with the accuracy that could be obtained by always predicting the majority class (ie, Yes CIPN-f or CIPN-h), referred to as the no information rate.

### Contribution of Functional Assessments in NeuroDetect Models

The contribution of each functional assessment (eg, natural walk, Romberg stance, and finger tapping) to NeuroDetect Model classification was assessed by building and evaluating exploratory models that included only a single functional assessment and models that included all but 1 of the functional assessments using a similar process as that used to build the NeuroDetect Models. The AUC of each exploratory model was compared with the NeuroDetect Models using all functional assessments by a 1-sided bootstrap method with 400 permutations.

### Comparisons With CIPN20 Models and Combined Models

To compare the performance between functional assessments and PROs to detect CIPN-f and CIPN-h, a similar process was used to build models for CIPN-f and CIPN-h that included either CIPN20 items only (CIPN20 Models) or the combination of CIPN20 items and NeuroDetect features (Combined Models). A 2-sided bootstrap method with 400 permutations was used to compare the AUCs of these models for detecting CIPN-f or CIPN-h at the end of treatment.

To evaluate the performance of each model for detecting CIPN at earlier time points during treatment, data collected from NeuroDetect Models, CIPN20 Models, and Combined Models at months 0 (M0, before day 0), 1 (M1, from day 0 to day 29), 2 (M2, from day 30 to day 59), and 3 (M3, day 60+) were used, and the AUC for detecting CIPN-f and CIPN-h was calculated and compared using the same 2-sided bootstrap method. The earliest time point a model could differentiate patients with and those without CIPN is estimated based on the first consecutive day that there was a statistically significant association between the model classification and CIPN diagnosis using a Fisher exact test. Multiple comparisons for testing each day throughout treatment were accounted for using the Benjamini and Hochberg false discovery rate.

### Ethical Considerations

This study was approved by the University of Michigan IRBMed (HUM00171478). All participants completed oral informed consent before downloading NeuroDetect and then completed comprehensive written informed consent within NeuroDetect. All data were collected within secure databases accessible only to the study team. Clinical data were collected within REDCap (Research Electronic Data Capture; Vanderbilt University) and NeuroDetect data were collected within the secure data environment provided by Medable. Patients were compensated US $50 if they completed at least 3 NeuroDetect assessments and the clinical neurological assessment, otherwise, they did not receive compensation and were replaced in the study. No identifying information for individual participants is included in this manuscript.

## Results

### Patient Enrollment and CIPN Data Collection

Of the 85 eligible patients who indicated interest in participating, a total of 66 patients enrolled and completed CIPN20 and functional assessments at baseline and at least once during treatment, and 45 completed a neurological examination at the end of treatment (Multimedia Appendix 2). Among these 45 patients, 24 had CIPN-f and 29 had CIPN-h (Table 1). On average, each patient performed each gait and balance assessment 7.5 times, each manual dexterity assessment 7.4 times, and CIPN20 7.4 times during treatment. Most patients (40/45, 80%) completed NeuroDetect assessments and CIPN20 questionnaires every 3 weeks. After data cleaning, the walking and standing data had 567 unique samples throughout treatment, including 70 samples at the end of treatment, and the finger tapping and hole-peg test data had 318 samples throughout treatment, including 41 samples at the end of treatment. A total of 333 CIPN20 questionnaires were collected including 45 at the end of treatment.

**Table 1:** Clinical characteristics of the 45 patients included in the analyses.

Characteristic			Statistical value
Age (year), mean (SD)			48 (15)
**Sex, n (%)**			
	Female	40 (89)	
	Male	5 (11)	
**Self-reported race, n (%)**			
	White	38 (84)	
	Black	4 (9)	
	Asian	2 (4)	
	Other	1 (2)	
**Tumor type, n (%)**			
	Breast	40 (89)	
	Colorectal	5 (11)	
**Neurotoxic chemotherapy treatment, n (%)**			
	Taxane	26 (58)	
	Platinum	13 (29)	
	Taxane and platinum combination	8 (18)	
Neurotoxic chemotherapy treatment duration (week), mean (SD)			11.5 (4.6)
**Treatment adjustment, n (%)**			
	Dose delay	9 (20)	
	Dose decrease	13 (29)	
	Switch to nab-paclitaxel	2 (4)	
**CIPN^a^ from neuromuscular specialist assessment, n (%)**			
	CIPN in the feet (CIPN-f)	24 (53)	
	CIPN in the hands (CIPN-h)	29 (64)	
NeuroDetect gait and balance assessments per patient, mean (SD)			6.9 7.5 (9.2)
NeuroDetect manual dexterity assessments per patient, mean (SD)			7.4 (8.9)
CIPN20^b^ assessments per patient, mean (SD)			7.4 (8.1)

^a^CIPN: chemotherapy-induced peripheral neuropathy.

^b^CIPN20: 20-item scale.

### NeuroDetect Models for Detecting CIPN at the End of Treatment

**Figure 1:**
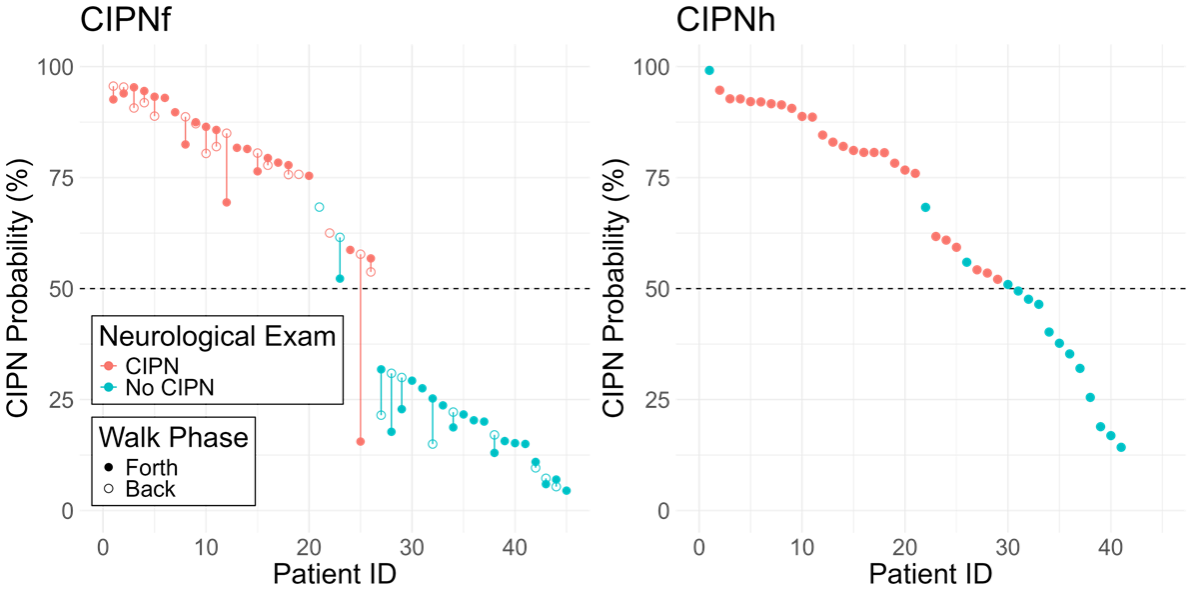
Predicted chemotherapy-induced peripheral neuropathy probability of NeuroDetect models at the end of treatment. CIPN: chemotherapy-induced peripheral neuropathy; CIPNf: chemotherapy-induced peripheral neuropathy in the feet; CIPNh: chemotherapy-induced peripheral neuropathy in the hands.

From the 3700 features generated from the 6 functional assessments (Table S1 in Multimedia Appendix 1), minimum redundancy maximum relevance selected 300 gait and balance features and 20 manual dexterity features, of which elastic net selected 182 gait and balance features and 19 manual dexterity features to be included in the final NeuroDetect Models of CIPN-f and CIPN-h, respectively (Tables S2 and S3 in Multimedia Appendix 1). The NeuroDetect Model accuracy was significantly higher than no information rate for CIPN-f (80% vs 53%, *P*=.02, Table 2, Figure 1) but not CIPN-h (82% vs 64%, *P*=.18).

**Table 2:** End-of-treatment chemotherapy-induced peripheral neuropathy detection performance by NeuroDetect, CIPN20, and Combined Models.

Performance metric	CIPN-f^a^ Models				CIPN-h^b^ Models		
	NeuroDetect	CIPN20^c^	Combined	NeuroDetect		CIPN20	Combined
						
Area under curve	0.84	0.76	0.94	0.68		0.56	0.86
Accuracy	0.80	0.69	0.85	0.82		0.58	0.73
Accuracy versus NIR^d^, *P* value^e^	.02^f^	.20	.005^f^	.18		.82	.39

^a^CIPN-f: chemotherapy-induced peripheral neuropathy in the feet.

^b^CIPN-h: chemotherapy-induced peripheral neuropathy in the hands.

^c^CIPN20: 20-item scale.

^d^NIR: no information rate.

^e^*P* value testing accuracy of each Model versus the no information rate, which is the accuracy obtained by always predicting the majority class.

^f^Significance level *P*<.05.

All data were collected within a prospective observational study of 45 patients with cancer receiving neurotoxic chemotherapy treatment who completed NeuroDetect functional assessments during treatment and a clinical neurological assessment at the end of treatment. The NeuroDetect models were used to estimate the likelihood (y-axis) that each patient had CIPN-f (left panel) and CIPN-h (right panel) at the end of neurotoxic chemotherapy treatment. Patients are reordered in descending order of the strength of the CIPN prediction probability from NeuroDetect. There was strong concordance between these estimated probabilities and the clinical diagnosis from the clinical neurological examination; red circles indicate patients who were diagnosed with CIPN, and blue circles indicate patients who were not. Closed and open circles indicate the 2 walking phases, forth and back, respectively, which were connected by a line for each patient. There was strong agreement between CIPN-f probability estimated between the 2 walking phases in all except a single patient.

The final NeuroDetect Models are enriched for features from Romberg stance (40.2% of total variable importance in CIPN-f) and finger tapping (84.7% of total variable importance in CIPN-h, Table S4 in Multimedia Appendix 1) assessments. The 3 most important CIPN-f features are all rolling rotations from tandem walk and Romberg stance assessments (Table S2 in Multimedia Appendix 1). The most important CIPN-h feature is the autocorrelation of the nondominant hand-tapping interval during the finger-tapping assessment (Table S3 in Multimedia Appendix 1). In CIPN-f detection, the model built using only features from Romberg stance had the best performance and had higher AUC than the model built using all functional assessments (AUC=98% vs 83.8%, *P*=.04, Table S5 in Multimedia Appendix 1). Similarly, the detection performance of the CIPN-f model declined most when removing Romberg Stance features (Table S6 in Multimedia Appendix 1), indicating that Romberg stance had the highest contribution to CIPN-f detection. Neither finger tapping nor hole-peg test had a significant independent contribution to CIPN-h classification (Tables S5 and S6 in Multimedia Appendix 1).

### Comparisons Between NeuroDetect, CIPN20, and Combined Models

**Figure 2:**
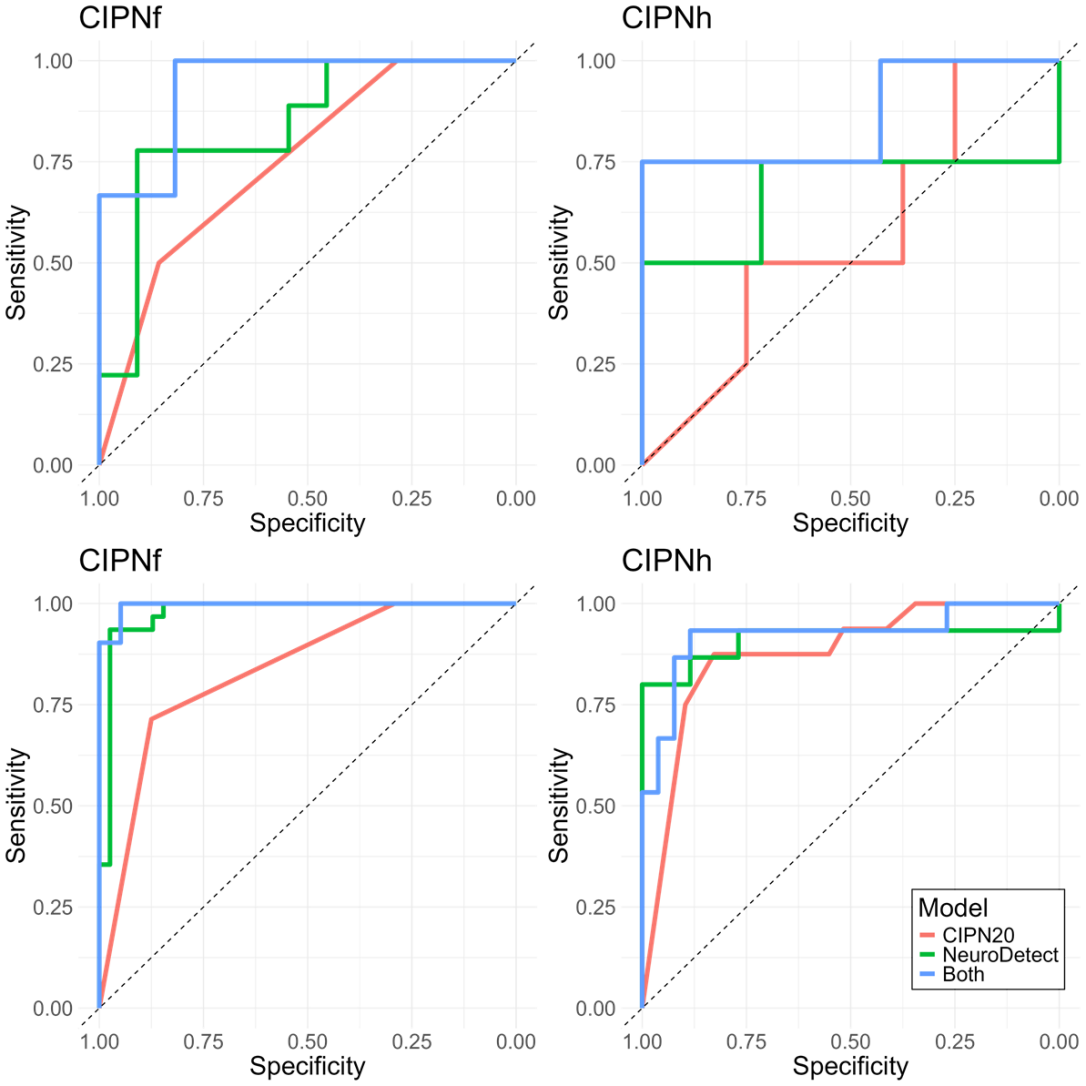
Receiver operating characteristic curves for chemotherapy-induced peripheral neuropathy detection at the end of treatment. CIPN20: 20-item scale; CIPNf: chemotherapy-induced peripheral neuropathy in the feet; CIPNh: chemotherapy-induced peripheral neuropathy in the hands.

In exploratory analyses comparing end-of-treatment CIPN detection between models, NeuroDetect Models had numerically, but not statistically, superior performance to CIPN20 Models in both CIPN-f (AUC=84% vs 76%, *P*=.55, Table 2 and Table S7 in Multimedia Appendix 1) and CIPN-h (AUC=68% vs 56%, *P*=.67). Combined Models numerically, but not statistically, outperformed either independently (Figure 2, Table S7 in Multimedia Appendix 1).

All data were collected within a prospective observational study of 45 patients with cancer receiving neurotoxic chemotherapy treatment who completed NeuroDetect functional assessments during treatment and a clinical neurological assessment at the end of treatment. Models built using only the features generated from the functional assessments conducted within NeuroDetect (NeuroDetect Models), only from the patient-reported CIPN20 questionnaire items (CIPN20 Models), or the combination of both (Combined Models) were compared for their accuracy in detecting CIPN-f and CIPN-h at the end of treatment. CIPN-f and CIPN-h are based on a clinical neurological assessment. The top 2 panels present the sensitivity and specificity of the CIPN-f (left) and CIPN-h (right) models in only the unbiased independent testing data set (30% of the entire data set, not used in model training). The bottom 2 panels present similar results for the entire data set, including both the training and testing data. Colored lines indicate results from the NeuroDetect Models (green), CIPN20 Models (red), and Combined Models (blue). NeuroDetect Models had a good AUC with balanced sensitivity and specificity. In CIPN-f classification, the NeuroDetect and Combined Models had a greater AUC than the CIPN20 Model.

**Figure 3:**
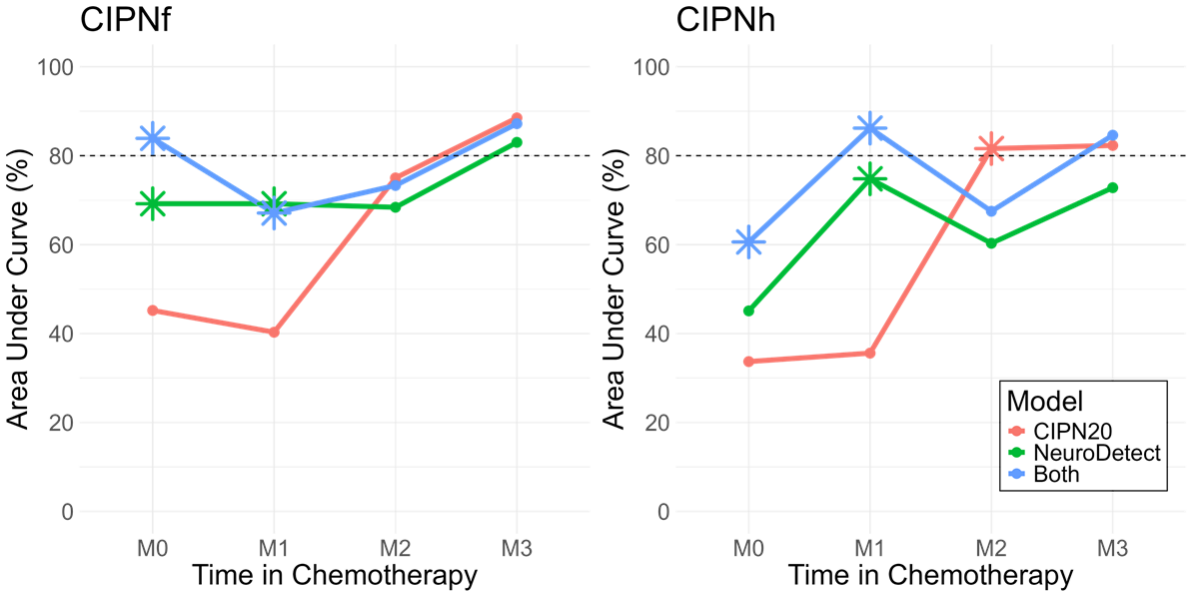
Area under the curve of models over the course of treatment. CIPN20: 20-item scale; CIPNf: chemotherapy-induced peripheral neuropathy in the feet; CIPNh: chemotherapy-induced peripheral neuropathy in the hands.

Models were then compared on their ability to detect CIPN at earlier time points using the patient data at months 0, 1, 2, and 3 of treatment. NeuroDetect and Combined models were numerically, and in most cases statistically, superior to CIPN20 models at M0 (pretreatment) and at M1, but the models were similarly accurate by month 2 (Figure 3 and Table S7 in Multimedia Appendix 1).

All data were collected within a prospective observational study of 45 patients with cancer receiving neurotoxic chemotherapy treatment who completed NeuroDetect functional assessments during treatment and a clinical neurological assessment at the end of treatment. Data before treatment (M0) and during months 1 (from day 0 to day 29), 2 (from day 30 to day 59), and 3 (from day 60 to day 89) of treatment were used to evaluate the performance of the CIPN detection models. Models were generated using only the NeuroDetect objective assessments (green), only the patient-reported CIPN20 questionnaires (red), and a Combined Model using both (blue). The data from each model collected before and throughout treatment were used to estimate model performance through AUC. The true CIPN-f and CIPN-h were based on the clinical neurological assessment conducted at the end of treatment. Asterisk dots indicate significantly higher AUC than at least 1 of the other 2 models by a bootstrap method. NeuroDetect and Combined Models were superior to CIPN20 Models before treatment (M0) and at M1, but models generally had nonsignificantly different performance after M2. These analyses suggest that CIPN detection before and early in treatment can be improved by using NeuroDetect functional assessments in addition to patient-reported questionnaires.

**Figure 4:**
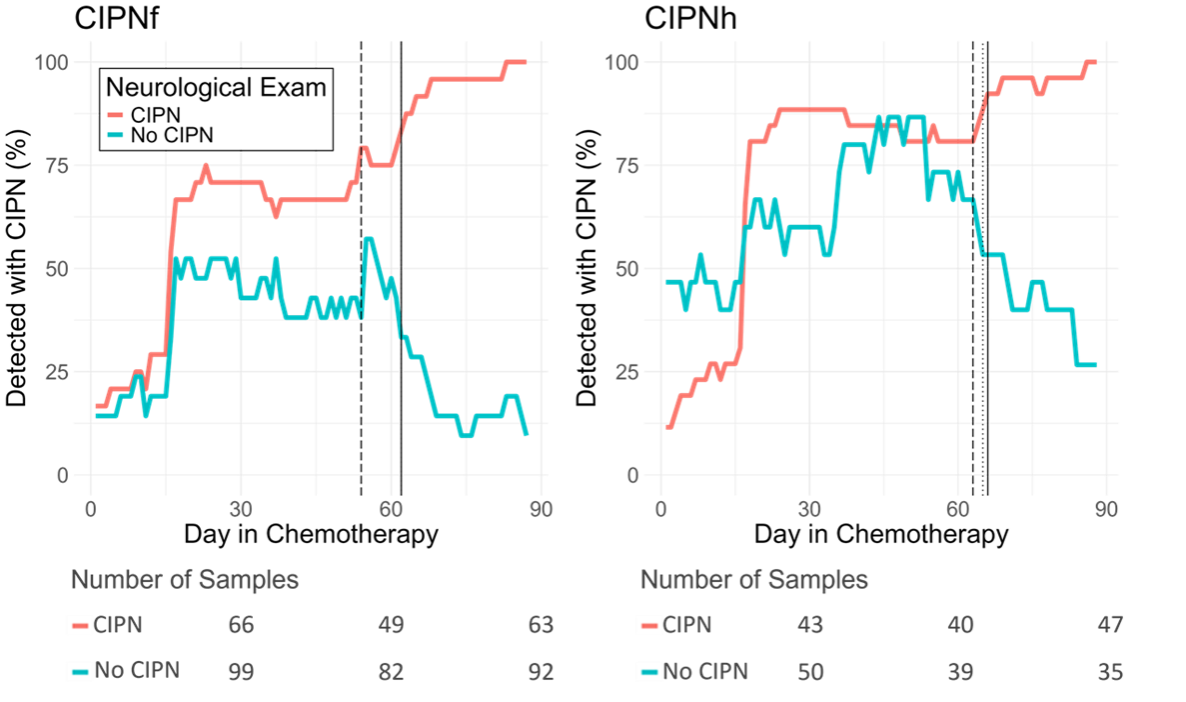
Percentage of patients detected with chemotherapy-induced peripheral neuropathy over time. CIPN: chemotherapy-induced peripheral neuropathy; CIPNf: chemotherapy-induced peripheral neuropathy in the feet; CIPNh: chemotherapy-induced peripheral neuropathy in the hands.

Finally, analyses were conducted to determine at what point in treatment the NeuroDetect and other Models could discriminate between patients with and those without CIPN. For CIPN-f, the NeuroDetect Model could statistically discriminate between patients with and those without CIPN on day 62 (Figure 4), which was numerically though not statistically later than the CIPN20 Model and similar to the Combined Model (indicated in Figure 4, raw data not shown). For CIPN-h, the NeuroDetect Model reached significance at day 65 (Figure 4), which was not statistically different from that of the CIPN20 or Combined Models.

All data were collected within a prospective observational study of 45 patients with cancer receiving neurotoxic chemotherapy treatment who completed NeuroDetect functional assessments during treatment and a clinical neurological assessment at the end of treatment. Data collected during treatment were used to estimate the earliest time point the Models created from only the NeuroDetect functional assessments could statistically differentiate patients with and those without CIPN. Similar analyses were conducted for the models generated from only the CIPN20 patient-reported questionnaires and the combination of both data types (Combined Model) for comparison (raw data not shown). Red lines indicate the group of patients diagnosed with CIPN from neurological examinations conducted at the end of treatment and blue lines indicate the group not diagnosed with CIPN. For CIPN-f, the NeuroDetect Model reached statistical significance at day 62 (*P*=.003, solid vertical line), which is the same day that CIPN discrimination was significant for the Combined model (dotted vertical line not visible due to overlay with solid line). The CIPN20 Model discrimination was significant at day 54 (dashed vertical line), though this difference was not statistically significant. For CIPN-h, the NeuroDetect Model reached statistical significance at day 66 (solid vertical line), which was not statistically different from the day the CIPN20 (day 63, dashed vertical line) and Combined (day 65, dotted vertical line) Models reached statistical significance.

## Discussion

### Principal Results

Tremendous advancements in the development of smartphone and wearable sensor–based technology in health care enable various apps relating to disease and treatment [10], including monitoring gait, balance, and manual dexterity changes in Parkinson disease [11]. Like Parkinson disease, CIPN is associated with changes in gait and balance, and reductions in manual strength and dexterity. Detecting CIPN is essential to prevent its progression to debilitating symptoms that can irreversibly diminish function and quality of life [4,5]. The current approach to CIPN detection, which relies on patient’s reporting symptoms during appointments, has limitations that are not addressed by currently available clinic-based objective assessment or PRO subjective assessment strategies. Our previous NeuroDetect V1.0 cross-sectional study revealed evidence that a single timepoint remote, smartphone sensor–based functional assessments after neurotoxic chemotherapy treatment can differentiate patients with and those without CIPN [13]. Building upon our previous study, the objective of this NeuroDetect V2.0 longitudinal study was to determine the feasibility of completing longitudinal app-based functional assessments that emulate certain aspects of the neurological examination that can detect CIPN during neurotoxic treatment. In addition, we conducted exploratory comparisons of functional assessments collected via NeuroDetect V2.0 with CIPN20 questionnaires. The results demonstrated the feasibility of integrating subjective and objective CIPN assessment into a smartphone app for remote, longitudinal CIPN monitoring.

### Comparison With Previous Work

In this longitudinal observational cohort study, the NeuroDetect Model effectively distinguished patients with and those without end-of-treatment CIPN-f based on gold-standard clinical assessment by neuromuscular specialists, while maintaining an acceptable false discovery rate. The features contributing the most in the final CIPN-f NeuroDetect Model were rolling rotation, which is intertwined with the side-to-side swaying movement features from balance assessments. These features during the eyes closed period of the Romberg stance assessment showed the greatest contribution to CIPN-f detection. Furthermore, 2 previous studies used smartphone or smart device sensors to monitor CIPN [13,29]. One study did not detect any correlation between CIPN and a small set of gait and balance features [29]. Alternatively, in our cross-sectional study of NeuroDetct V1.0, a side-to-side swaying acceleration pattern during a normal walk assessment indicated diminished balance in patients with CIPN [13]. Side-to-side swaying features were also identified in studies with open-eye and closed-eye balancing assessments using commercial or self-designed wearable devices and software [30-32] or other clinic-based balance assessments [12,14]. Previous studies have also reported shorter step lengths in patients with CIPN [4,13,32]. This analysis could not be conducted since NeuroDetect V2.0 does not estimate step length.

The CIPN-h NeuroDetect Model was not able to distinguish patients with and those without end-of-treatment CIPN-h, which might suggest a more sensitive functional assessment is needed. Our NeuroDetect V1.0 and V2.0 studies are the only attempts to our knowledge to objectively evaluate manual dexterity using a remote app-based strategy [13]. The hole-peg test is an on-screen assessment that is supposed to mimic the 9-hole peg test, which is often used to assess manual dexterity, in which patients physically pick up pegs and stick them into a hole on a board [20]. In our cross-sectional study of NeuroDetect V1.0, features from the hole-peg test were indicative of CIPN. However, user feedback indicated that the hole-peg test was confusing to operate on a screen [13], which was also reported by patients within poststudy interviews conducted in this longitudinal study (data not included). In the longitudinal study of NeuroDetect V2.0, the newly added finger-tapping assessment was more informative than the hole-peg test, but the overall performance remained poor. Finger tapping is also used as an objective manual strength assessment in neurological disorders and hereditary peripheral neuropathy [33,34], although its use in CIPN has not been validated to our knowledge. Adding other screen-compatible manual dexterity assessments, such as line tracing, might improve the ability of smartphone sensor–based assessments to detect diminished manual dexterity indicative of CIPN-h [35]. One potential challenge will be to develop manual dexterity assessments that are specific for CIPN symptoms without the interference of other chemotherapy toxicities, such as cognitive impairment [36].

In exploratory model comparisons, combining both NeuroDetect features and CIPN20 items together in a Combined Model numerically outperformed each Model individually early in treatment. This finding indicates that integration of sensory-based functional assessments with PRO may improve CIPN detection, which is consistent with previous research and statements that support combining subjective and objective CIPN assessments to improve CIPN detection [37]. The potential benefits of a combined strategy are further supported by our exploratory analysis, which suggested that CIPN20 differentiates between patients with and those without CIPN 2-8 days earlier than NeuroDetect. Currently available hybrid assessment strategies can be time-consuming and require specialized equipment and trained professionals [37] and have not been demonstrated to improve CIPN detection compared with using only PROs [32,38]. On the other hand, NeuroDetect might provide a convenient option for objectively monitoring CIPN by a smartphone [39] that can be completed at home, where the patient is comfortable, and at a time that is convenient, with no added cost for purchasing equipment since most patients own a smartphone. Objective functional assessments can accurately detect CIPN without being influenced by a patient’s tolerance for living with CIPN symptoms [40].

Although there is no effective treatment for CIPN, detecting CIPN can prompt early interventions, such as physical therapy referrals, chemotherapy dose reductions, or drug switching, to prevent severe and irreversible CIPN [41]. Interestingly, our results indicate that pretreatment functional NeuroDetect assessments, but not PRO assessments, may be able to identify patients who are likely to experience CIPN during treatment. This is consistent with other evidence that pre-existing neuropathy, such as that resulting from hereditary neuropathy conditions, diabetes, or advanced age, is a major risk factor for treatment-limiting CIPN [1,41], perhaps due to treatment-related exacerbation of subclinical symptoms. If this is validated in future studies, NeuroDetect may be a particularly convenient screening tool to identify potential participants for interventional trials of CIPN prevention or treatment, including approaches like acupuncture, cryo-compression, and exercise therapy [42], which could ameliorate the disparity in CIPN among non-White patients [43].

### Limitations

This study advanced the development of app-based functional CIPN assessment by using a longitudinal study design and gold-standard clinical neurological history and examination for CIPN diagnosis. It also used contemporary machine learning modeling for complex multidimensional data and conducted comparative and integrative analyses to evaluate the benefits of using NeuroDetect alone or in combination with PRO. Despite these strengths, several limitations are worth considering. First, this was a small cohort with many features extracted. Rigorous data processing and model training were used to prevent overfitting and provide evidence of feasibility. Much larger clinical studies are needed to validate a NeuroDetect app and CIPN detection model for clinical use and to further investigate the benefits of adding functional NeuroDetect assessment to PRO CIPN monitoring. Second, in longitudinal analysis, the model classification during treatment could only be compared with the neurological diagnoses at the end of treatment, rather than a direct comparison with the CIPN at that moment. Collecting longitudinal objective CIPN outcomes, perhaps using a hybrid assessment such as the Total Neuropathy Score [37], is crucial for future studies attempting to develop real-time CIPN detection or outcome trajectory prediction. Third, remote functional assessments inherently rely on patient understanding and adherence to instructions, but the nonsupervised process and nonstandardized environments can introduce undesirable variability and missingness in data. This can be mitigated by simplified tasks, clear instructions, and regular reminders and is a trade-off we have accepted to gain convenience of remote CIPN detection without specialized equipment. Finally, given the small patient cohort, we did not make any attempt to conduct subgroup analyses or adjust for other important clinical variables such as age or neurotoxic chemotherapy regimen (eg, taxane vs platinum). Our analysis did not detect statistically significant differences in age in patients who were CIPN-h or CIPN-f cases versus those who were controls (data not shown). However, since age likely affects patient’s performance of functional assessments, age will be explored in detail for potential incorporation into the NeuroDetect models built within larger patient cohorts. It is also possible that other clinical consequences of treatment, such as general fatigue, would contribute to worsening performance on functional tasks, though these data were not collected within the study so this could not be explored.

### Conclusions

Our findings demonstrate the feasibility of integrating subjective and objective CIPN assessment into a smartphone app for remote, longitudinal CIPN monitoring. Future work will build NeuroDetect V3.0 and generate validated CIPN-f and CIPN-h detection models in much larger patient cohorts with longitudinal CIPN assessment. Eventually, prospective randomized controlled trials will be needed to investigate whether app-based CIPN monitoring via NeuroDetect improves CIPN detection and treatment outcomes, such as reducing persistent CIPN, in patients with cancer.

## Appendix

Multimedia Appendix 1 Additional Tables.

Multimedia Appendix 2 Number of patients enrolled and analyzed in the analyses.

## Abbreviations

AUC: area under the curve
CIPN: chemotherapy-induced peripheral neuropathy
CIPN20: 20-item scale
CIPN-f: chemotherapy-induced peripheral neuropathy in the feet
CIPN-h: chemotherapy-induced peripheral neuropathy in the hand
EORTC: European Organization for Research and Treatment of Cancer
PRO: patient-reported outcome
QLQ: Quality of Life Questionnaire
REDCap: Research Electronic Data Capture

## Data Availability

All data and code will be made available upon reasonable request to the corresponding author. Due to the preliminary nature of these findings, these models are not intended to be used to detect CIPN or guide treatment in clinical practice. Researchers interested in contributing to the ongoing development of NeuroDetect should contact the corresponding author.

## References

[1] Seretny M, Currie GL, Sena ES, Ramnarine S, Grant R, MacLeod MR, Colvin LA, Fallon M (2014). Incidence, prevalence, and predictors of chemotherapy-induced peripheral neuropathy: a systematic review and meta-analysis. Pain.

[2] Miltenburg NC, Boogerd W (2014). Chemotherapy-induced neuropathy: a comprehensive survey. Cancer Treat Rev.

[3] Quasthoff S, Hartung HP (2002). Chemotherapy-induced peripheral neuropathy. J Neurol.

[4] Winters-Stone KM, Horak F, Jacobs PG, Trubowitz P, Dieckmann NF, Stoyles S, Faithfull S (2017). Falls, functioning, and disability among women with persistent symptoms of chemotherapy-induced peripheral neuropathy. J Clin Oncol.

[5] Bandos H, Melnikow J, Rivera DR, Swain SM, Sturtz K, Fehrenbacher L, Wade JL, Brufsky AM, Julian TB, Margolese RG, McCarron EC, Ganz PA (2018). Long-term peripheral neuropathy in breast cancer patients treated with adjuvant chemotherapy: NRG oncology/NSABP B-30. J Natl Cancer Inst.

[6] Knoerl R, Smith EML, Han A, Doe A, Scott K, Berry DL (2019). Characterizing patient-clinician chemotherapy-induced peripheral neuropathy assessment and management communication approaches. Patient Educ Couns.

[7] Park SB, Goldstein D, Krishnan AV, Lin CS, Friedlander ML, Cassidy J, Koltzenburg M, Kiernan MC (2013). Chemotherapy-induced peripheral neurotoxicity: a critical analysis. CA Cancer J Clin.

[8] Cavaletti G, Cornblath DR, Merkies ISJ, Postma TJ, Rossi E, Frigeni B, Alberti P, Bruna J, Velasco R, Argyriou AA, Kalofonos HP, Psimaras D, Ricard D, Pace A, Galiè E, Briani C, Dalla Torre C, Faber CG, Lalisang RI, Boogerd W, Brandsma D, Koeppen S, Hense J, Storey D, Kerrigan S, Schenone A, Fabbri S, Valsecchi MG, Mazzeo A, Pace A, Pessino A, Schenone A, Toscano A, Argyriou AA, Brouwer B, Frigeni B, Piras B, Briani C, Dalla Torre C, Dominguez Gonzalez C, Faber CG, Tomasello C, Binda D, Brandsma D, Cortinovis D, Psimaras D, Ricard D, Storey D, Cornblath DR, Galiè E, Lindeck Pozza E, Rossi E, Vanhoutte EK, Lanzani F, Pastorelli F, Altavilla G, Cavaletti G, Granata G, Kalofonos HP, Ghignotti I, Merkies ISJ, Bruna J, Hense J, Heimans JJ, Mattavelli L, Padua L, Reni L, Bakkers M, Boogerd M, Campagnolo M, Cazzaniga M, Eurelings M, Leandri M, Lucchetta M, Penas Prado M, Russo M, Valsecchi MG, Piatti ML, Alberti P, Bidoli P, Grant R, Plasmati R, Velasco R, Lalisang RI, Meijer RJ, Fabbri S, Dorsey SG, Galimberti S, Kerrigan S, Koeppen S, Postma TJ, Boogerd W, Grisold W, CI-PeriNomS Group (2013). The chemotherapy-induced peripheral neuropathy outcome measures standardization study: from consensus to the first validity and reliability findings. Ann Oncol.

[9] Salgado TM, Quinn CS, Krumbach EK, Wenceslao I, Gonzalez M, Reed HL, Syverson JG, Etz RS, Vangipuram K, Barker MR, Henry NL, Farris KB, Hertz DL (2020). Reporting of paclitaxel-induced peripheral neuropathy symptoms to clinicians among women with breast cancer: a qualitative study. Support Care Cancer.

[10] Guo CC, Chiesa PA, de Moor C, Fazeli MS, Schofield T, Hofer K, Belachew S, Scotland A (2022). Digital devices for assessing motor functions in mobility-impaired and healthy populations: systematic literature review. J Med Internet Res.

[11] Tripathi S, Malhotra A, Qazi M, Chou J, Wang F, Barkan S, Hellmers N, Henchcliffe C, Sarva H (2022). Clinical review of smartphone applications in parkinson's disease. Neurologist.

[12] Mantovani E, Demrozi F, Hertz DL, Turetta C, Ferro O, Argyriou AA, Pravadelli G, Tamburin S (2022). Wearables, sensors, and smart devices for the detection and monitoring of chemotherapy-induced peripheral neurotoxicity: systematic review and directions for future research. J Peripher Nerv Syst.

[13] Chen CS, Kim J, Garg N, Guntupalli H, Jagsi R, Griggs JJ, Sabel M, Dorsch MP, Callaghan BC, Hertz DL (2021). Chemotherapy-induced peripheral neuropathy detection via a smartphone app: cross-sectional pilot study. JMIR Mhealth Uhealth.

[14] Campbell G, Skubic MA (2018). Balance and gait impairment: sensor-based assessment for patients with peripheral neuropathy. Clin J Oncol Nurs.

[15] Knoerl R, Gray E, Stricker C, Mitchell SA, Kippe K, Smith G, Dudley WN, Lavoie Smith EM (2017). Electronic versus paper-pencil methods for assessing chemotherapy-induced peripheral neuropathy. Support Care Cancer.

[16] Developer A (2023). ResearchKit. Apple Developer Documentation.

[17] Findling O, van der Logt R, Nedeltchev K, Achtnichts L, Allum JHJ (2018). A comparison of balance control during stance and gait in patients with inflammatory and non-inflammatory polyneuropathy. PLoS One.

[18] Richardson JK (2002). The clinical identification of peripheral neuropathy among older persons. Arch Phys Med Rehabil.

[19] Missaoui B, Thoumie P (2013). Balance training in ataxic neuropathies. Effects on balance and gait parameters. Gait Posture.

[20] Yancosek KE, Howell D (2009). A narrative review of dexterity assessments. J Hand Ther.

[21] Postma TJ, Aaronson NK, Heimans JJ, Muller MJ, Hildebrand JG, Delattre JY, Hoang-Xuan K, Lantéri-Minet M, Grant R, Huddart R, Moynihan C, Maher J, Lucey R, EORTC Quality of Life Group (2005). The development of an EORTC quality of life questionnaire to assess chemotherapy-induced peripheral neuropathy: the QLQ-CIPN20. Eur J Cancer.

[22] Dyck PJ, Albers JW, Andersen H, Arezzo JC, Biessels G, Bril V, Feldman EL, Litchy WJ, O'Brien PC, Russell JW, Toronto Expert Panel on Diabetic Neuropathy (2011). Diabetic polyneuropathies: update on research definition, diagnostic criteria and estimation of severity. Diabetes Metab Res Rev.

[23] Tesfaye S, Vileikyte L, Rayman G, Sindrup SH, Perkins BA, Baconja M, Vinik AI, Boulton AJM, Toronto Expert Panel on Diabetic Neuropathy (2011). Painful diabetic peripheral neuropathy: consensus recommendations on diagnosis, assessment and management. Diabetes Metab Res Rev.

[24] Snyder P, Tummalacherla M, Perumal T, Omberg L (2020). mhealthtools: a modular R package for extracting features from mobile and wearable sensor data. JOSS.

[25] De Jay N, Papillon-Cavanagh S, Olsen C, El-Hachem N, Bontempi G, Haibe-Kains B (2013). mRMRe: an R package for parallelized mRMR ensemble feature selection. Bioinformatics.

[26] Friedman J, Hastie T, Tibshirani R (2010). Regularization paths for generalized linear models via coordinate descent. J Stat Softw.

[27] Kuhn M (2008). Building predictive models in R using the caret package. J. Stat. Soft.

[28] Tay JK, Narasimhan B, Hastie T (2023). Elastic net regularization paths for all generalized linear models. J Stat Softw.

[29] Jacobs PG, Hanaway P, Leitschuh J, Condon J, Rajhbeharrysingh U, Mosquera-Lopez C, Guidarelli C, Winters-Stone K (2018). Design and evaluation of a portable smart-phone based peripheral neuropathy test platform. Annu Int Conf IEEE Eng Med Biol Soc.

[30] Fino PC, Horak FB, El-Gohary M, Guidarelli C, Medysky ME, Nagle SJ, Winters-Stone KM (2019). Postural sway, falls, and self-reported neuropathy in aging female cancer survivors. Gait Posture.

[31] Zahiri M, Chen KM, Zhou H, Nguyen H, Workeneh BT, Yellapragada SV, Sada YH, Schwenk M, Najafi B (2019). Using wearables to screen motor performance deterioration because of cancer and chemotherapy-induced peripheral neuropathy (CIPN) in adults - toward an early diagnosis of CIPN. J Geriatr Oncol.

[32] Monfort SM, Pan X, Patrick R, Ramaswamy B, Wesolowski R, Naughton MJ, Loprinzi CL, Chaudhari AMW, Lustberg MB (2017). Gait, balance, and patient-reported outcomes during taxane-based chemotherapy in early-stage breast cancer patients. Breast Cancer Res Treat.

[33] Gopal A, Hsu WY, Allen DD, Bove R (2022). Remote assessments of hand function in neurological disorders: systematic review. JMIR Rehabil Assist Technol.

[34] Prada V, Robbiano G, Mennella G, Hamedani M, Bellone E, Grandis M, Schenone A, Zuccarino R (2020). Validation of a new hand function outcome measure in individuals with charcot-marie-tooth disease. J Peripher Nerv Syst.

[35] Rabah A, Le Boterff Q, Carment L, Bendjemaa N, Térémetz M, Dupin L, Cuenca M, Mas J, Krebs M, Maier MA, Lindberg PG (2022). A novel tablet-based application for assessment of manual dexterity and its components: a reliability and validity study in healthy subjects. J Neuroeng Rehabil.

[36] Milutinovic B, Singh AK (2022). Editorial: cognitive impairment and peripheral neuropathy from chemotherapy: molecular mechanisms and therapeutic approaches. Front Mol Biosci.

[37] Park SB, Alberti P, Kolb NA, Gewandter JS, Schenone A, Argyriou AA (2019). Overview and critical revision of clinical assessment tools in chemotherapy-induced peripheral neurotoxicity. J Peripher Nerv Syst.

[38] McCrary JM, Goldstein D, Trinh T, Timmins HC, Li T, Friedlander M, Bosco A, Harrison M, Maier N, O'Neill S, Park SB (2019). Optimizing clinical screening for chemotherapy-induced peripheral neuropathy. J Pain Symptom Manage.

[39] Fischer F, Kleen S (2021). Possibilities, problems, and perspectives of data collection by mobile apps in longitudinal epidemiological studies: scoping review. J Med Internet Res.

[40] Hertz DL (2019). Concerns regarding use of patient-reported outcomes in biomarker studies of chemotherapy-induced peripheral neuropathy. Pharmacogenomics J.

[41] Hertz DL, Childs DS, Park SB, Faithfull S, Ke Y, Ali NT, McGlown SM, Chan A, Grech LB, Loprinzi CL, Ruddy KJ, Lustberg M (2021). Patient-centric decision framework for treatment alterations in patients with chemotherapy-induced peripheral neuropathy (CIPN). Cancer Treat Rev.

[42] Loprinzi CL, Lacchetti C, Bleeker J, Cavaletti G, Chauhan C, Hertz DL, Kelley MR, Lavino A, Lustberg MB, Paice JA, Schneider BP, Lavoie Smith EM, Smith ML, Smith TJ, Wagner-Johnston N, Hershman DL (2020). Prevention and management of chemotherapy-induced peripheral neuropathy in survivors of adult cancers: ASCO guideline update. J Clin Oncol.

[43] Schneider BP, Zhao F, Ballinger TJ, Garcia SF, Shen F, Virani S, Cella D, Bales C, Jiang G, Hayes L, Miller N, Srinivasiah J, Stringer-Reasor EM, Chitalia A, Davis AA, Makower DF, Incorvati J, Simon MA, Mitchell EP, DeMichele A, Miller KD, Sparano JA, Wagner LI, Wolff AC (2024). ECOG-ACRIN EAZ171: prospective validation trial of germline predictors of taxane-induced peripheral neuropathy in black women with early-stage breast cancer. J Clin Oncol.

