# Supplementary material for: Remote Monitoring of Chemotherapy-Induced Peripheral Neuropathy by the NeuroDetect iOS App: Observational Cohort Study of Patients With Cancer
Source: J Med Internet Res. 2025 Feb 5;27:e65615. doi: 10.2196/65615 (PMC11840369; doi:10.2196/65615)
Supplement: Multimedia Appendix 1 [file jmir_v27i1e65615_app1.docx]

**Table S1.** Number of features generated from each NeuroDetect functional assessment.

|  | Gait and Balance | | Balance | | Manual Dexterity | |
| --- | --- | --- | --- | --- | --- | --- |
|  | Natural Walk | Tandem Walk | Tandem Stance | Romberg Stance | Finger Tapping | Hole-peg Test |
| Acceleration | 516 | 516 | 258 | 516 | NA | NA |
| Rotation | 516 | 516 | 258 | 516 | NA | NA |
| Screen touch | NA | NA | NA | NA | 80 | 8 |
| Total | 1032 | 1032 | 516 | 1032 | 80 | 8 |

NA: not applicable.

**Table S2.** Gait and balance features included in the NeuroDetect CIPN-f model.

| Feature^a^ | Average mRMR Score | Elastic Net Coefficient | Elastic Net Variable Importance |
| --- | --- | --- | --- |
| walk_tandem_mode2_rotation_y_f_energy_band_7.5 | 0.12 | 0.11 | 100 |
| stance_romberg_open_rotation_y_f_energy_band_3 | 0.01 | -0.09 | 83 |
| walk_tandem_mode2_rotation_y_f_energy_band_7 | 0.04 | 0.09 | 83 |
| stance_romberg_closed_rotation_z_f_energy_band_2.5 | 0.05 | 0.09 | 76 |
| walk_natural_mode1_acceleration_y_f_energy_band_3.5 | 0.05 | -0.08 | 74 |
| walk_tandem_mode2_rotation_y_f_energy_band_8 | 0.07 | 0.08 | 70 |
| walk_tandem_mode1_acceleration_z_t_med | 0.05 | 0.08 | 69 |
| stance_romberg_closed_rotation_z_f_skewness | 0.02 | -0.08 | 67 |
| walk_natural_mode1_rotation_x_f_ewt_renyi_entropy | 0.06 | -0.07 | 62 |
| stance_romberg_open_rotation_y_f_energy_band_2.5 | 0.05 | -0.07 | 61 |
| walk_natural_mode1_rotation_x_f_ewt_shannon_entropy | 0.04 | -0.07 | 61 |
| walk_natural_mode1_rotation_x_f_ewt_simpson_entropy | 0.03 | -0.07 | 57 |
| stance_romberg_open_rotation_x_f_energy_band_8 | 0.05 | 0.07 | 57 |
| stance_romberg_open_acceleration_x_f_ewt_tsallis_entropy | 0.03 | 0.06 | 56 |
| walk_tandem_mode1_rotation_z_t_rms | 0.09 | -0.06 | 54 |
| walk_tandem_mode1_rotation_z_t_sd | 0.06 | -0.06 | 54 |
| walk_natural_mode1_acceleration_y_f_energy_band_3 | 0.02 | -0.06 | 52 |
| stance_tandem_rotation_x_f_energy_band_12.5 | 0.02 | 0.06 | 52 |
| walk_natural_mode1_acceleration_x_t_kurtosis | 0.11 | 0.06 | 51 |
| walk_natural_mode1_acceleration_z_t_kurtosis | 0.05 | 0.06 | 50 |
| stance_romberg_open_rotation_x_f_energy_band_9 | 0.04 | 0.06 | 49 |
| stance_tandem_rotation_x_f_energy_band_11.5 | 0.03 | 0.06 | 49 |
| stance_tandem_rotation_z_t_mu | 0.04 | 0.05 | 48 |
| stance_romberg_closed_acceleration_y_f_energy_band_3.5 | 0.06 | 0.05 | 48 |
| walk_tandem_mode1_rotation_z_t_min | 0.04 | 0.05 | 48 |
| walk_tandem_mode1_acceleration_y_f_sd | <0.001 | 0.05 | 48 |
| stance_romberg_open_rotation_y_f_energy_band_13 | 0.04 | 0.05 | 47 |
| stance_romberg_closed_rotation_z_f_energy_band_4.5 | 0.008 | 0.05 | 47 |
| walk_tandem_mode2_rotation_y_f_energy_band_8.5 | 0.03 | 0.05 | 46 |
| stance_romberg_open_rotation_y_f_energy_band_1.5 | 0.14 | -0.05 | 46 |
| walk_tandem_mode1_rotation_y_t_max | 0.08 | -0.05 | 44 |
| walk_natural_mode1_rotation_z_f_energy_band_4.5 | 0.03 | 0.05 | 43 |
| stance_romberg_closed_rotation_x_t_complexity | 0.12 | -0.05 | 41 |
| stance_romberg_open_rotation_y_f_energy_band_12.5 | 0.05 | 0.05 | 41 |
| walk_natural_mode2_rotation_x_f_energy_band_22 | 0.01 | 0.05 | 40 |
| walk_tandem_mode2_acceleration_z_f_ewt_tsallis_entropy | 0.01 | 0.04 | 39 |
| walk_natural_mode1_acceleration_z_f_energy_band_4.5 | 0.03 | -0.04 | 39 |
| walk_tandem_mode1_rotation_z_t_rugosity | 0.07 | -0.04 | 39 |
| stance_romberg_open_rotation_y_f_energy_band_13.5 | 0.08 | 0.04 | 38 |
| stance_romberg_closed_rotation_y_t_skewness | 0.05 | -0.04 | 36 |
| stance_romberg_open_rotation_x_f_energy_band_2 | 0.008 | -0.04 | 36 |
| stance_romberg_open_rotation_x_f_energy_band_7.5 | 0.03 | 0.04 | 35 |
| walk_tandem_mode2_acceleration_z_t_Q25 | 0.03 | 0.04 | 35 |
| walk_natural_mode1_acceleration_x_t_skewness | 0.03 | 0.04 | 34 |
| walk_tandem_mode1_rotation_y_f_ewt_simpson_entropy | 0.04 | -0.04 | 34 |
| walk_tandem_mode1_rotation_y_f_ewt_tsallis_entropy | 0.02 | -0.04 | 34 |
| stance_romberg_closed_acceleration_y_f_energy_band_1 | 0.12 | -0.04 | 34 |
| stance_romberg_closed_rotation_z_f_energy_band_12 | 0.04 | 0.04 | 33 |
| walk_tandem_mode1_rotation_z_t_energy | 0.16 | -0.04 | 33 |
| stance_romberg_open_rotation_y_f_energy_band_8.5 | 0.03 | 0.04 | 33 |
| walk_tandem_mode1_rotation_z_f_energy_band_9 | 0.009 | 0.04 | 33 |
| walk_tandem_mode1_acceleration_z_t_mu | 0.01 | 0.04 | 33 |
| stance_romberg_closed_acceleration_y_t_skewness | 0.02 | 0.04 | 32 |
| walk_tandem_mode1_rotation_y_f_ewt_shannon_entropy | 0.02 | -0.04 | 32 |
| walk_tandem_mode1_acceleration_y_t_rugosity | 0.06 | -0.04 | 32 |
| stance_romberg_open_acceleration_z_f_skewness | 0.06 | 0.04 | 32 |
| walk_tandem_mode1_rotation_y_t_rms | 0.05 | -0.04 | 31 |
| stance_tandem_rotation_y_f_ewt_simpson_entropy | 0.03 | -0.03 | 30 |
| stance_romberg_closed_rotation_z_f_mod | 0.16 | 0.03 | 30 |
| walk_tandem_mode1_acceleration_y_f_energy_band_4 | 0.008 | -0.03 | 29 |
| stance_romberg_open_rotation_z_f_mod | 0.006 | -0.03 | 29 |
| stance_romberg_open_rotation_y_f_energy_band_8 | 0.05 | 0.03 | 29 |
| walk_natural_mode1_acceleration_y_f_energy_band_4 | 0.001 | -0.03 | 29 |
| stance_tandem_rotation_y_f_ewt_renyi_entropy | 0.05 | -0.03 | 28 |
| walk_tandem_mode1_rotation_z_t_Q75 | 0.05 | -0.03 | 28 |
| walk_tandem_mode1_rotation_y_f_ewt_renyi_entropy | 0.01 | -0.03 | 28 |
| walk_natural_mode1_acceleration_x_f_energy_band_5.5 | 0.04 | -0.03 | 28 |
| stance_tandem_rotation_x_f_energy_band_7.5 | 0.02 | 0.03 | 27 |
| stance_romberg_closed_rotation_z_f_energy_band_11.5 | 0.03 | 0.03 | 27 |
| walk_tandem_mode1_acceleration_y_f_energy_band_1 | 0.006 | 0.03 | 27 |
| walk_tandem_mode1_rotation_z_t_med | 0.04 | -0.03 | 27 |
| stance_romberg_closed_acceleration_z_f_energy_band_9.5 | 0.04 | -0.03 | 27 |
| walk_natural_mode2_rotation_x_t_kurtosis | 0.01 | 0.03 | 27 |
| stance_romberg_closed_rotation_z_f_energy_band_12.5 | 0.06 | 0.03 | 26 |
| walk_tandem_mode1_rotation_y_t_energy | 0.07 | -0.03 | 26 |
| walk_natural_mode1_rotation_y_t_kurtosis | 0.03 | 0.03 | 25 |
| stance_romberg_closed_acceleration_z_f_energy_band_9 | 0.07 | -0.03 | 25 |
| stance_romberg_open_rotation_y_f_energy_band_2 | 0.08 | -0.03 | 25 |
| stance_tandem_acceleration_z_f_energy_band_8 | 0.03 | -0.03 | 25 |
| walk_tandem_mode1_rotation_y_t_Q75 | 0.10 | -0.03 | 24 |
| stance_romberg_closed_rotation_y_f_energy_band_13 | 0.03 | 0.03 | 24 |
| stance_romberg_open_rotation_y_f_energy_band_14 | 0.06 | 0.03 | 24 |
| walk_natural_mode1_rotation_y_f_energy_band_19.5 | 0.02 | 0.03 | 24 |
| walk_natural_mode2_rotation_y_f_energy_band_19 | 0.002 | -0.03 | 24 |
| stance_romberg_open_rotation_z_f_ewt_renyi_entropy | 0.04 | 0.03 | 24 |
| stance_romberg_open_rotation_z_f_energy_band_7.5 | 0.002 | 0.03 | 24 |
| walk_tandem_mode2_acceleration_z_f_energy_band_9.5 | 0.01 | 0.03 | 23 |
| stance_romberg_closed_rotation_z_f_energy_band_1 | 0.03 | -0.03 | 23 |
| stance_romberg_closed_acceleration_z_f_energy_band_10 | 0.03 | -0.03 | 23 |
| walk_natural_mode1_rotation_y_f_energy_band_20 | 0.06 | 0.03 | 23 |
| walk_tandem_mode1_rotation_z_t_rough | 0.04 | -0.03 | 22 |
| stance_romberg_open_acceleration_z_f_ewt_permutation_entropy | 0.05 | -0.02 | 22 |
| walk_natural_mode1_acceleration_x_f_energy_band_6 | 0.01 | -0.02 | 21 |
| stance_romberg_closed_rotation_y_f_energy_band_13.5 | 0.04 | 0.02 | 21 |
| stance_romberg_closed_rotation_y_f_skewness | 0.04 | -0.02 | 20 |
| stance_romberg_closed_acceleration_z_f_ewt_simpson_entropy | 0.04 | -0.02 | 20 |
| stance_romberg_open_rotation_x_t_tk_energy_mu | 0.02 | -0.02 | 20 |
| stance_romberg_open_rotation_y_f_energy_band_11.5 | 0.03 | 0.02 | 20 |
| walk_natural_mode2_rotation_y_f_energy_band_18 | 0.008 | -0.02 | 19 |
| stance_romberg_closed_rotation_x_t_mu | 0.12 | -0.02 | 19 |
| walk_tandem_mode1_rotation_z_t_tk_energy_mu | 0.07 | -0.02 | 19 |
| stance_romberg_open_rotation_y_f_energy_band_1 | 0.08 | -0.02 | 19 |
| stance_romberg_open_acceleration_z_f_ewt_renyi_entropy | 0.03 | -0.02 | 18 |
| stance_romberg_open_rotation_x_f_energy_band_1.5 | 0.03 | -0.02 | 18 |
| walk_natural_mode2_rotation_z_t_med | <0.001 | -0.02 | 18 |
| walk_natural_mode2_rotation_z_f_ewt_tsallis_entropy | 0.03 | -0.02 | 18 |
| stance_tandem_rotation_y_f_ewt_tsallis_entropy | 0.05 | -0.02 | 17 |
| stance_romberg_closed_acceleration_z_f_energy_band_8.5 | 0.05 | -0.02 | 17 |
| walk_natural_mode1_acceleration_x_f_energy_band_10.5 | 0.007 | -0.02 | 17 |
| walk_natural_mode2_rotation_y_f_energy_band_16 | 0.01 | -0.02 | 17 |
| walk_tandem_mode2_rotation_y_f_energy_band_4.5 | 0.01 | -0.02 | 16 |
| stance_romberg_closed_rotation_y_f_energy_band_8.5 | 0.02 | 0.02 | 16 |
| stance_romberg_open_acceleration_z_f_kurtosis | 0.09 | 0.02 | 16 |
| stance_romberg_closed_rotation_y_f_energy_band_11.5 | 0.02 | 0.02 | 15 |
| walk_tandem_mode1_rotation_z_f_energy_band_13 | 0.02 | 0.02 | 15 |
| walk_natural_mode1_acceleration_z_t_Q75 | 0.03 | -0.02 | 15 |
| stance_romberg_open_rotation_z_f_energy_band_2.5 | 0.05 | 0.02 | 15 |
| walk_tandem_mode1_acceleration_z_f_energy_band_5.5 | 0.002 | -0.02 | 15 |
| walk_natural_mode2_acceleration_x_t_skewness | 0.01 | -0.02 | 14 |
| walk_natural_mode2_rotation_z_f_ewt_shannon_entropy | 0.02 | -0.02 | 14 |
| stance_romberg_closed_acceleration_y_f_energy_band_7.5 | 0.002 | 0.02 | 14 |
| walk_natural_mode1_rotation_y_f_energy_band_22.5 | 0.04 | 0.02 | 14 |
| walk_natural_mode1_rotation_y_f_energy_band_18.5 | 0.03 | 0.02 | 14 |
| walk_tandem_mode1_rotation_y_f_energy_band_3.5 | 0.02 | -0.02 | 14 |
| stance_romberg_open_acceleration_y_f_ewt_tsallis_entropy | 0.04 | 0.02 | 13 |
| stance_romberg_open_acceleration_z_f_max | 0.04 | 0.02 | 13 |
| stance_romberg_open_rotation_x_f_energy_band_7 | 0.02 | 0.01 | 13 |
| walk_tandem_mode1_acceleration_y_t_tk_energy_mu | 0.05 | -0.01 | 13 |
| walk_natural_mode2_acceleration_z_t_kurtosis | 0.05 | 0.01 | 13 |
| walk_natural_mode1_rotation_z_f_energy_band_22 | 0.04 | 0.01 | 13 |
| walk_tandem_mode1_acceleration_y_t_rough | 0.05 | -0.01 | 12 |
| stance_tandem_rotation_y_f_energy_band_2 | 0.06 | -0.01 | 12 |
| stance_romberg_open_rotation_z_f_skewness | 0.02 | -0.01 | 12 |
| walk_tandem_mode1_acceleration_y_f_shannon_entropy | 0.004 | 0.01 | 11 |
| walk_natural_mode1_rotation_x_f_ewt_tsallis_entropy | 0.03 | -0.01 | 11 |
| stance_romberg_closed_rotation_y_f_kurtosis | 0.02 | -0.01 | 11 |
| walk_tandem_mode2_acceleration_x_f_ewt_renyi_entropy | 0.04 | 0.01 | 11 |
| stance_romberg_closed_rotation_y_f_energy_band_9 | 0.04 | 0.01 | 11 |
| walk_tandem_mode1_acceleration_x_t_mu | 0.02 | 0.01 | 11 |
| walk_natural_mode2_rotation_y_f_energy_band_15.5 | 0.001 | -0.01 | 10 |
| stance_tandem_acceleration_y_t_rough | 0.03 | 0.01 | 10 |
| stance_tandem_acceleration_y_f_energy_band_3 | 0.02 | -0.01 | 10 |
| stance_tandem_acceleration_y_f_energy_band_2.5 | 0.01 | -0.01 | 10 |
| stance_romberg_open_rotation_y_f_energy_band_10 | 0.07 | 0.01 | 10 |
| stance_tandem_rotation_y_f_energy_band_2.5 | 0.01 | -0.01 | 9 |
| stance_tandem_rotation_z_f_energy_band_7 | 0.01 | 0.01 | 9 |
| stance_romberg_closed_rotation_y_f_energy_band_9.5 | 0.03 | 0.01 | 9 |
| stance_romberg_open_rotation_x_f_energy_band_1 | 0.06 | -0.01 | 8 |
| stance_romberg_closed_acceleration_y_f_energy_band_4 | 0.05 | 0.009 | 8 |
| stance_romberg_open_acceleration_x_f_energy_band_1 | 0.03 | -0.009 | 8 |
| stance_tandem_acceleration_y_f_energy_band_3.5 | 0.005 | -0.009 | 8 |
| stance_romberg_open_rotation_z_f_energy_band_2 | 0.04 | 0.008 | 7 |
| walk_natural_mode1_acceleration_x_f_energy_band_3 | 0.01 | 0.008 | 7 |
| stance_tandem_acceleration_x_t_med | 0.06 | 0.008 | 7 |
| stance_romberg_open_acceleration_z_f_ewt_simpson_entropy | 0.02 | -0.008 | 7 |
| walk_natural_mode1_rotation_y_f_ewt_renyi_entropy | 0.008 | 0.008 | 7 |
| walk_natural_mode1_rotation_y_f_energy_band_23 | 0.05 | 0.007 | 7 |
| stance_romberg_closed_rotation_x_f_energy_band_6 | 0.01 | 0.007 | 6 |
| walk_tandem_mode1_rotation_z_f_energy_band_21.5 | 0.03 | 0.007 | 6 |
| stance_romberg_open_rotation_y_f_energy_band_7.5 | 0.02 | 0.007 | 6 |
| stance_romberg_closed_acceleration_y_f_energy_band_7 | 0.01 | 0.007 | 6 |
| stance_romberg_closed_rotation_z_f_ewt_simpson_entropy | 0.01 | 0.007 | 6 |
| walk_tandem_mode2_acceleration_x_f_ewt_tsallis_entropy | 0.04 | 0.005 | 5 |
| walk_natural_mode1_rotation_y_t_IQR | 0.03 | 0.005 | 5 |
| walk_natural_mode1_rotation_x_f_energy_band_3.5 | 0.006 | -0.004 | 4 |
| stance_romberg_open_rotation_z_f_energy_band_12 | 0.02 | 0.004 | 3 |
| stance_romberg_open_rotation_x_f_mod | 0.15 | 0.004 | 3 |
| walk_tandem_mode1_rotation_y_t_rough | 0.07 | -0.004 | 3 |
| walk_natural_mode1_acceleration_x_f_energy_band_3.5 | 0.02 | 0.003 | 3 |
| walk_tandem_mode2_acceleration_z_t_mu | 0.01 | 0.003 | 3 |
| stance_romberg_closed_rotation_x_t_Q75 | 0.07 | 0.003 | 2 |
| walk_tandem_mode1_acceleration_z_f_energy_band_8.5 | <0.001 | 0.002 | 2 |
| walk_natural_mode1_rotation_y_t_Q75 | 0.06 | -0.002 | 2 |
| walk_tandem_mode2_acceleration_y_f_energy_band_4 | 0.008 | -0.002 | 2 |
| stance_romberg_closed_acceleration_y_f_energy_band_4.5 | 0.03 | 0.001 | 1 |
| stance_romberg_closed_acceleration_y_f_energy_band_6.5 | 0.03 | 0.001 | 1 |
| walk_tandem_mode2_acceleration_y_f_energy_band_4.5 | 0.01 | -0.001 | 1 |
| walk_tandem_mode2_acceleration_z_t_skewness | 0.02 | 0.001 | 1 |
| walk_natural_mode1_rotation_y_t_Q25 | 0.03 | 0.001 | 1 |
| stance_romberg_open_acceleration_x_f_mod | 0.009 | 0.001 | 1 |
| walk_natural_mode1_acceleration_y_f_kurtosis | 0.004 | 0.001 | 1 |
| stance_tandem_rotation_z_f_energy_band_12.5 | 0.005 | 0 | 0 |
| stance_tandem_acceleration_x_t_kurtosis | 0.23 | 0 | 0 |
| stance_romberg_closed_rotation_x_f_mod | 0.21 | 0 | 0 |
| stance_romberg_closed_rotation_x_t_rugosity | 0.17 | 0 | 0 |
| stance_romberg_closed_rotation_x_t_tk_energy_mu | 0.15 | 0 | 0 |
| stance_romberg_open_rotation_y_t_mobility | 0.12 | 0 | 0 |
| stance_romberg_closed_rotation_y_f_mod | 0.10 | 0 | 0 |
| stance_tandem_acceleration_x_t_mod | 0.08 | 0 | 0 |
| stance_romberg_closed_rotation_y_t_rough | 0.07 | 0 | 0 |
| stance_romberg_closed_rotation_y_f_md | 0.07 | 0 | 0 |
| stance_romberg_closed_acceleration_y_f_mod | 0.07 | 0 | 0 |
| walk_tandem_mode2_acceleration_x_f_energy_band_1 | 0.06 | 0 | 0 |
| stance_romberg_open_rotation_y_f_IQR | 0.05 | 0 | 0 |
| stance_tandem_rotation_z_t_kurtosis | 0.05 | 0 | 0 |
| stance_romberg_closed_acceleration_y_f_Q25 | 0.05 | 0 | 0 |
| stance_tandem_acceleration_z_t_kurtosis | 0.05 | 0 | 0 |
| stance_tandem_rotation_y_t_max | 0.05 | 0 | 0 |
| stance_tandem_acceleration_x_t_tk_energy_mu | 0.04 | 0 | 0 |
| stance_romberg_open_rotation_x_f_min | 0.04 | 0 | 0 |
| stance_romberg_closed_rotation_x_t_IQR | 0.04 | 0 | 0 |
| stance_romberg_closed_rotation_x_t_rough | 0.04 | 0 | 0 |
| walk_natural_mode2_rotation_z_t_kurtosis | 0.04 | 0 | 0 |
| stance_romberg_closed_rotation_x_f_Q25 | 0.04 | 0 | 0 |
| walk_natural_mode2_acceleration_z_f_energy_band_5 | 0.04 | 0 | 0 |
| stance_romberg_closed_rotation_y_f_Q75 | 0.04 | 0 | 0 |
| walk_natural_mode1_acceleration_z_f_energy_band_3 | 0.03 | 0 | 0 |
| walk_natural_mode1_rotation_y_t_energy | 0.03 | 0 | 0 |
| stance_romberg_closed_acceleration_y_f_energy_band_1.5 | 0.03 | 0 | 0 |
| stance_romberg_open_rotation_x_f_md | 0.03 | 0 | 0 |
| stance_romberg_closed_rotation_x_f_energy_band_5 | 0.03 | 0 | 0 |
| walk_natural_mode1_acceleration_x_t_Q25 | 0.03 | 0 | 0 |
| stance_romberg_closed_acceleration_z_f_energy_band_5 | 0.03 | 0 | 0 |
| stance_tandem_rotation_z_f_mod | 0.03 | 0 | 0 |
| walk_natural_mode1_acceleration_x_t_rough | 0.03 | 0 | 0 |
| stance_romberg_open_rotation_x_f_Q25 | 0.03 | 0 | 0 |
| stance_romberg_open_rotation_z_f_max | 0.03 | 0 | 0 |
| walk_tandem_mode1_rotation_y_t_mu | 0.03 | 0 | 0 |
| stance_romberg_closed_rotation_x_f_kurtosis | 0.03 | 0 | 0 |
| stance_romberg_closed_rotation_z_t_mu | 0.03 | 0 | 0 |
| stance_romberg_open_acceleration_x_f_Q25 | 0.03 | 0 | 0 |
| stance_romberg_open_rotation_y_t_rough | 0.03 | 0 | 0 |
| stance_romberg_closed_rotation_x_t_Q25 | 0.03 | 0 | 0 |
| stance_tandem_rotation_y_f_energy_band_1.5 | 0.03 | 0 | 0 |
| stance_romberg_open_acceleration_z_t_kurtosis | 0.03 | 0 | 0 |
| stance_tandem_rotation_z_t_range | 0.03 | 0 | 0 |
| stance_romberg_closed_acceleration_z_f_ewt_permutation_entropy | 0.03 | 0 | 0 |
| walk_tandem_mode2_acceleration_z_f_ewt_simpson_entropy | 0.02 | 0 | 0 |
| stance_romberg_closed_acceleration_x_f_Q25 | 0.02 | 0 | 0 |
| stance_romberg_closed_acceleration_x_t_Q75 | 0.02 | 0 | 0 |
| stance_romberg_closed_rotation_y_f_energy_band_14.5 | 0.02 | 0 | 0 |
| walk_natural_mode1_acceleration_x_t_Q75 | 0.02 | 0 | 0 |
| walk_natural_mode1_rotation_z_f_max | 0.02 | 0 | 0 |
| walk_natural_mode1_acceleration_z_f_energy_band_2.5 | 0.02 | 0 | 0 |
| walk_natural_mode1_rotation_y_f_energy_band_24 | 0.02 | 0 | 0 |
| stance_romberg_open_acceleration_y_f_energy_band_7.5 | 0.02 | 0 | 0 |
| stance_romberg_closed_acceleration_x_f_ewt_tsallis_entropy | 0.02 | 0 | 0 |
| stance_romberg_open_acceleration_z_f_energy_band_1.5 | 0.02 | 0 | 0 |
| stance_romberg_closed_acceleration_x_f_energy_band_1 | 0.02 | 0 | 0 |
| stance_romberg_closed_acceleration_y_f_energy_band_5.5 | 0.02 | 0 | 0 |
| walk_tandem_mode1_rotation_y_t_med | 0.02 | 0 | 0 |
| stance_romberg_closed_acceleration_x_f_mod | 0.02 | 0 | 0 |
| walk_natural_mode1_acceleration_x_f_energy_band_16 | 0.02 | 0 | 0 |
| stance_romberg_closed_acceleration_y_t_IQR | 0.02 | 0 | 0 |
| stance_romberg_open_acceleration_y_f_energy_band_3.5 | 0.02 | 0 | 0 |
| stance_romberg_closed_rotation_x_f_md | 0.02 | 0 | 0 |
| stance_romberg_open_rotation_x_t_mu | 0.02 | 0 | 0 |
| walk_natural_mode1_acceleration_z_f_energy_band_3.5 | 0.02 | 0 | 0 |
| stance_romberg_closed_rotation_x_f_skewness | 0.02 | 0 | 0 |
| stance_romberg_open_rotation_x_f_energy_band_6.5 | 0.02 | 0 | 0 |
| stance_romberg_open_rotation_x_t_Q75 | 0.02 | 0 | 0 |
| walk_natural_mode2_acceleration_z_t_skewness | 0.02 | 0 | 0 |
| walk_natural_mode2_acceleration_y_t_range | 0.02 | 0 | 0 |
| walk_natural_mode1_acceleration_x_f_ewt_renyi_entropy | 0.02 | 0 | 0 |
| walk_natural_mode1_acceleration_x_t_IQR | 0.02 | 0 | 0 |
| stance_romberg_closed_rotation_z_f_md | 0.02 | 0 | 0 |
| stance_romberg_closed_rotation_y_t_med | 0.02 | 0 | 0 |
| stance_tandem_acceleration_y_f_energy_band_24.5 | 0.02 | 0 | 0 |
| walk_tandem_mode2_rotation_z_t_mu | 0.01 | 0 | 0 |
| walk_natural_mode2_rotation_y_f_energy_band_4.5 | 0.01 | 0 | 0 |
| walk_tandem_mode1_rotation_y_f_energy_band_3 | 0.01 | 0 | 0 |
| stance_romberg_closed_rotation_x_f_energy_band_5.5 | 0.01 | 0 | 0 |
| walk_natural_mode1_acceleration_y_t_rough | 0.01 | 0 | 0 |
| stance_romberg_closed_rotation_y_f_shannon_entropy | 0.01 | 0 | 0 |
| walk_natural_mode1_rotation_y_t_rugosity | 0.01 | 0 | 0 |
| walk_natural_mode1_acceleration_z_f_ewt_permutation_entropy | 0.01 | 0 | 0 |
| walk_tandem_mode1_rotation_y_t_kurtosis | 0.01 | 0 | 0 |
| walk_natural_mode2_rotation_y_f_energy_band_5.5 | 0.01 | 0 | 0 |
| stance_romberg_open_rotation_z_f_energy_band_10.5 | 0.01 | 0 | 0 |
| walk_tandem_mode1_rotation_y_f_energy_band_1 | 0.01 | 0 | 0 |
| walk_natural_mode2_acceleration_z_f_ewt_simpson_entropy | 0.01 | 0 | 0 |
| stance_romberg_open_rotation_y_f_ewt_tsallis_entropy | 0.01 | 0 | 0 |
| walk_natural_mode1_rotation_z_t_tk_energy_mu | 0.009 | 0 | 0 |
| walk_natural_mode1_acceleration_y_t_Q25 | 0.008 | 0 | 0 |
| walk_natural_mode2_rotation_y_f_energy_band_5 | 0.007 | 0 | 0 |
| walk_natural_mode2_rotation_x_t_med | 0.007 | 0 | 0 |
| stance_romberg_closed_rotation_x_f_energy_band_6.5 | 0.007 | 0 | 0 |
| walk_natural_mode1_rotation_y_t_tk_energy_mu | 0.006 | 0 | 0 |
| walk_natural_mode2_rotation_x_t_Q25 | 0.006 | 0 | 0 |
| walk_natural_mode2_acceleration_x_f_energy_band_5.5 | 0.006 | 0 | 0 |
| walk_natural_mode2_acceleration_x_t_med | 0.005 | 0 | 0 |
| walk_natural_mode2_rotation_y_f_energy_band_6 | 0.005 | 0 | 0 |
| stance_tandem_acceleration_y_t_med | 0.005 | 0 | 0 |
| stance_tandem_rotation_z_f_energy_band_9.5 | 0.005 | 0 | 0 |
| stance_romberg_open_acceleration_z_f_energy_band_8.5 | 0.004 | 0 | 0 |
| walk_natural_mode1_acceleration_z_f_ewt_renyi_entropy | 0.004 | 0 | 0 |
| walk_natural_mode2_acceleration_y_t_energy | 0.004 | 0 | 0 |
| stance_romberg_closed_acceleration_z_t_kurtosis | 0.004 | 0 | 0 |
| walk_tandem_mode1_acceleration_y_t_med | 0.004 | 0 | 0 |
| stance_tandem_rotation_y_f_energy_band_3 | 0.003 | 0 | 0 |
| stance_romberg_open_rotation_y_f_mod | 0.002 | 0 | 0 |
| walk_natural_mode1_acceleration_x_t_tk_energy_mu | 0.002 | 0 | 0 |
| stance_romberg_closed_rotation_y_f_energy_band_5 | 0.002 | 0 | 0 |
| walk_natural_mode2_acceleration_z_f_energy_band_2 | 0.002 | 0 | 0 |
| walk_natural_mode1_rotation_x_f_IQR | 0.001 | 0 | 0 |
| walk_tandem_mode2_acceleration_y_f_energy_band_3 | 0.001 | 0 | 0 |
| walk_natural_mode2_acceleration_z_f_ewt_renyi_entropy | 0.001 | 0 | 0 |
| walk_natural_mode2_acceleration_y_t_kurtosis | <0.001 | 0 | 0 |
| walk_natural_mode1_acceleration_y_f_ewt_simpson_entropy | <0.001 | 0 | 0 |
| stance_romberg_closed_rotation_z_f_ewt_renyi_entropy | <0.001 | 0 | 0 |

^a^The full definition of features can be found in the manual of mhealthtools package.

**Table S3.** Manual dexterity features included in the NeuroDetect CIPN-h model.

| Feature^a^ | Average mRMR Score | Elastic Net Coefficient | Elastic Net Variable Importance |
| --- | --- | --- | --- |
| finger_tap_nondominant_interval_autocorrelation2 | 0.15 | 0.42 | 100 |
| finger_tap_nondominant_drift_middle_kurtosis | 0.14 | -0.31 | 73 |
| finger_tap_nondominant_drift_middle_max | 0.08 | -0.29 | 69 |
| finger_tap_dominant_drift_index_med | 0.02 | -0.23 | 55 |
| finger_tap_nondominant_interval_kurtosis | 0.004 | -0.23 | 54 |
| finger_tap_dominant_interval_tk_energy | 0.09 | -0.21 | 51 |
| finger_peg_dominant_place_peg_time_mu | 0.03 | -0.20 | 47 |
| finger_tap_dominant_interval_min | 0.005 | 0.18 | 42 |
| finger_peg_nondominant_place_peg_time_sd | 0.02 | -0.17 | 40 |
| finger_peg_nondominant_remove_peg_time_mu | 0.07 | -0.14 | 33 |
| finger_tap_nondominant_interval_autocorrelation1 | 0.02 | -0.14 | 33 |
| finger_tap_nondominant_interval_tk_energy | 0.09 | 0.13 | 32 |
| finger_tap_dominant_drift_middle_range | 0.007 | 0.13 | 31 |
| finger_tap_nondominant_drift_middle_skewness | 0.02 | -0.13 | 30 |
| finger_tap_dominant_interval_iqr | 0.05 | -0.11 | 27 |
| finger_tap_dominant_drift_middle_mu | 0.01 | -0.11 | 26 |
| finger_tap_nondominant_interval_fatigue25 | -0.002 | -0.07 | 16 |
| finger_tap_nondominant_interval_range | 0.02 | 0.06 | 15 |
| finger_tap_nondominant_interval_mad | 0.05 | -0.05 | 13 |
| finger_tap_dominant_interval_cv | 0.04 | 0 | 0 |

^a^The full definition of features can be found in the manual of mhealthtools package.

**Table S4.** Contribution of each functional assessment to the NeuroDetect CIPN models.

|  | CIPN-f | | | | CIPN-h | |
| --- | --- | --- | --- | --- | --- | --- |
|  | Natural Walk | Tandem Walk | Tandem Stance | Romberg Stance | Finger Tapping | Hole-peg Test |
| Total number of features | 1032 | 1032 | 516 | 1032 | 80 | 8 |
| Number of features included in the CIPN model | 42 | 48 | 17 | 75 | 16 | 3 |
| Sum of variable importance | 1006 | 1384 | 352 | 1843 | 666 | 120 |
| Percentage of total variable importance | 21.9% | 30.2% | 7.7% | 40.2% | 84.7% | 15.3% |

**Table S5.** End-of-treatment CIPN detection performance by models including features from only a single NeuroDetect assessment.

|  | CIPN-f Models | | | | | | CIPN-h Models | | |
| --- | --- | --- | --- | --- | --- | --- | --- | --- | --- |
|  | All | Natural Walk | Tandem Walk | Tandem Stance | Romberg Stance | All | | Finger Tapping | Hole-peg Test |
| Area under curve | 0.84 | 0.73 | 0.60 | 0.84 | 0.98 | 0.68 | | 0.66 | 0.61 |
| Accuracy | 0.80 | 0.65 | 0.60 | 0.65 | 0.85 | 0.82 | | 0.75 | 0.64 |
| *P*-value of accuracy better than no informa-tion rate | 0.02 | 0.25 | 0.41 | 0.25 | 0.005 | 0.18 | | 0.39 | 0.63 |
| Kappa | 0.59 | 0.29 | 0.18 | 0.31 | 0.70 | 0.56 | | 0.31 | 0.00 |
| Mc-Nemar’s *P*-value | 0.62 | 1.00 | 0.72 | 0.45 | 1.00 | 0.48 | | 0.25 | 0.13 |
| Boot-strap *P*-value compared to all | NA | 0.83 | 1.00 | 0.50 | 0.04 | NA | | 0.53 | 0.65 |

NA, not applicable.

**Table S6.** End-of-treatment CIPN detection performance by models including features from all except one NeuroDetect assessment.

|  | CIPN-f Models | | | | | | CIPN-h Models | | |
| --- | --- | --- | --- | --- | --- | --- | --- | --- | --- |
|  | All | Exclud-ing Natural Walk | Exclud-ing Tandem Walk | Exclud-ing Tandem Stance | Exclud-ing Romberg Stance | All | | Exclud-ing Finger Tapping | Exclud-ing Hole-peg Test |
| Area under curve | 0.84 | 0.91 | 0.94 | 0.86 | 0.74 | 0.68 | | 0.61 | 0.66 |
| Accuracy | 0.80 | 0.80 | 0.90 | 0.80 | 0.75 | 0.82 | | 0.64 | 0.75 |
| *P*-value of accuracy better than no informa-tion rate | 0.02 | 0.02 | 0.001 | 0.02 | 0.06 | 0.18 | | 0.63 | 0.39 |
| Kappa | 0.59 | 0.60 | 0.80 | 0.59 | 0.49 | 0.56 | | 0.00 | 0.31 |
| Mc-Nemar’s *P*-value | 0.62 | 0.62 | 1.00 | 0.62 | 1.00 | 0.48 | | 0.13 | 0.25 |
| Boot-strap *P*-value compared to all | NA | 0.92 | 0.95 | 0.62 | 0.05 | NA | | 0.34 | 0.47 |

NA, not applicable.

**Table S7.** Bootstrap P-values comparing area under curve between models over the course of treatment.

|  | | M1 | M2 | M3 | End of Treatment |
| --- | --- | --- | --- | --- | --- |
| CIPN-f | NeuroDetect vs CIPN20 | **<0.001** | 0.33 | 0.25 | 0.55 |
|  | Combined vs CIPN20 | **<0.001** | 0.74 | 0.71 | 0.13 |
|  | Combined vs NeuroDetect | 0.62 | 0.33 | 0.22 | 0.16 |
| CIPN-h | NeuroDetect vs CIPN20 | **<0.001** | **0.008** | 0.20 | 0.67 |
|  | Combined vs CIPN20 | **<0.001** | **0.001** | 0.59 | 0.16 |
|  | Combined vs NeuroDetect | **0.03** | 0.27 | **0.04** | 0.15 |

M1, month 1 (from day 0 to day 29). M2, month 2 (from day 30 to day 59). M3, month 3+ (day 60+). Bold indicates *P*<.05.
