# Supplementary figures and images for: Remote Monitoring of Chemotherapy-Induced Peripheral Neuropathy by the NeuroDetect iOS App: Observational Cohort Study of Patients With Cancer
Source: J Med Internet Res. 2025 Feb 5;27:e65615. doi: 10.2196/65615 (PMC11840369; doi:10.2196/65615)

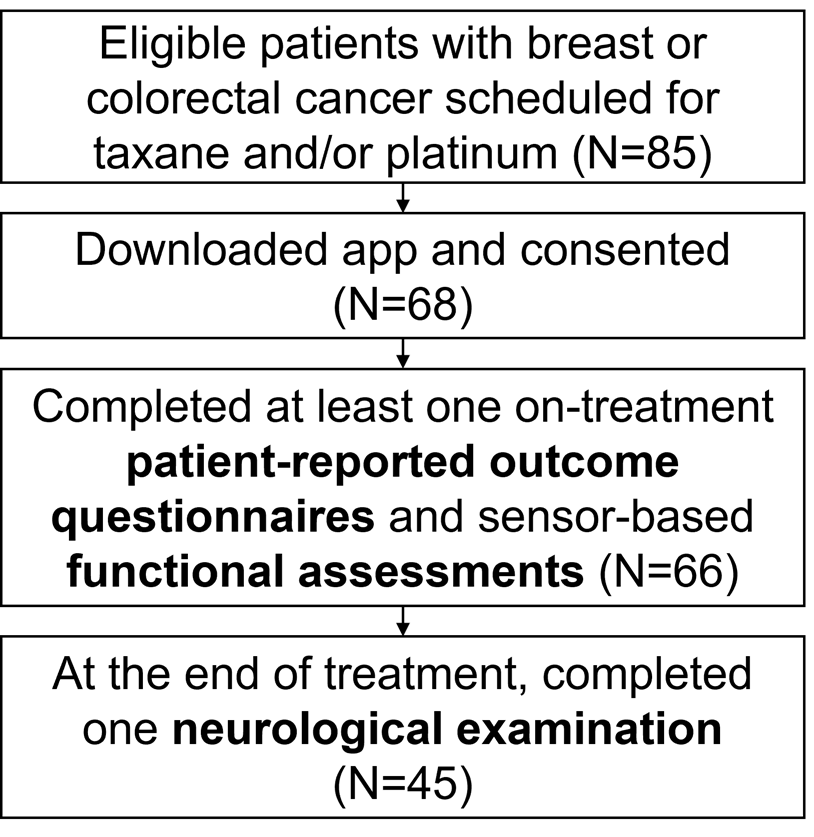

Supplement: Multimedia Appendix 2 [file jmir_v27i1e65615_app2.png]
